# Secondary plant metabolites as potent drug candidates against antimicrobial-resistant pathogens

**DOI:** 10.1007/s42452-022-05084-y

**Published:** 2022-07-08

**Authors:** Kadiatou Keita, Charles Darkoh, Florence Okafor

**Affiliations:** 1 Biological & Environmental Sciences, Alabama Agricultural & Mechanical University, Normal, AL 35762 USA; 2 Department of Epidemiology, School of Public Health, Center for Infectious Diseases, Human Genetics, and Environmental Sciences, University of Texas Health Science Center, Houston, TX 77030 USA; 3 MD Anderson Cancer Center UTHealth Graduate School of Biomedical Sciences, Microbiology and Infectious Diseases Program, Houston, TX 77030 USA

**Keywords:** Antimicrobial resistance, Plant metabolites, Plant secondary metabolites, Multidrug-resistant pathogens, Plant-based medical compounds, Anti-infective agents, Antibacterial drug screening

## Abstract

Antibiotic resistance is a major public health threat of the twenty-first century and represents an important risk to the global economy. Healthcare-associated infections mainly caused by drug-resistant bacteria are wreaking havoc in patient care worldwide. The spread of such pathogens limits the utility of available drugs and complicates the treatment of bacterial diseases. As a result, there is an urgent need for new drugs with mechanisms of action capable of curbing resistance. Plants synthesize and utilize various metabolic compounds to deter pathogens and predators. Utilizing these plant-based metabolites is a promising option in identifying novel bioactive compounds that could be harnessed to develop new potent antimicrobial drugs to treat multidrug-resistant pathogens. The purpose of this review is to highlight medicinal plants as important sources of novel antimicrobial agents that could be developed to help combat antimicrobial resistance.

## Introduction

Bacterial resistance to antibiotics constitutes one of the most important and urgent public health threats of the twenty-first century [1]. Infections caused by multidrug-resistant (MDR) pathogens are associated with increased mortality compared to those caused by drug-susceptible bacteria. The U.S. Center for Disease Control and Prevention (CDC) has designated antibiotic resistance as an important burden on the U.S. healthcare system, and over $20 billion are spent on treatment cost every year [2]. MDR pathogens are projected to cause about 300 million premature deaths worldwide and up to $100 trillion loss to the global economy by 2050 [3, 4].

Given the threat posed by drug-resistant bacteria, there is an urgent need for novel compounds with diverse mechanisms of action capable of limiting antimicrobial resistance. Secondary plant metabolites are one of the unexplored sources of antimicrobial agents in nature. It is estimated that less than 1% of the global tropical plant species have been screened for pharmaceutical applications [5] and investigated phytochemically [6]. Given the spread of multidrug-resistant pathogens and the dwindling number of available antibiotics, there is renewed interest in utilizing plant-based sources to identify potent novel antimicrobial agents.

### The declining potency of antibiotics

**Fig. 1:**
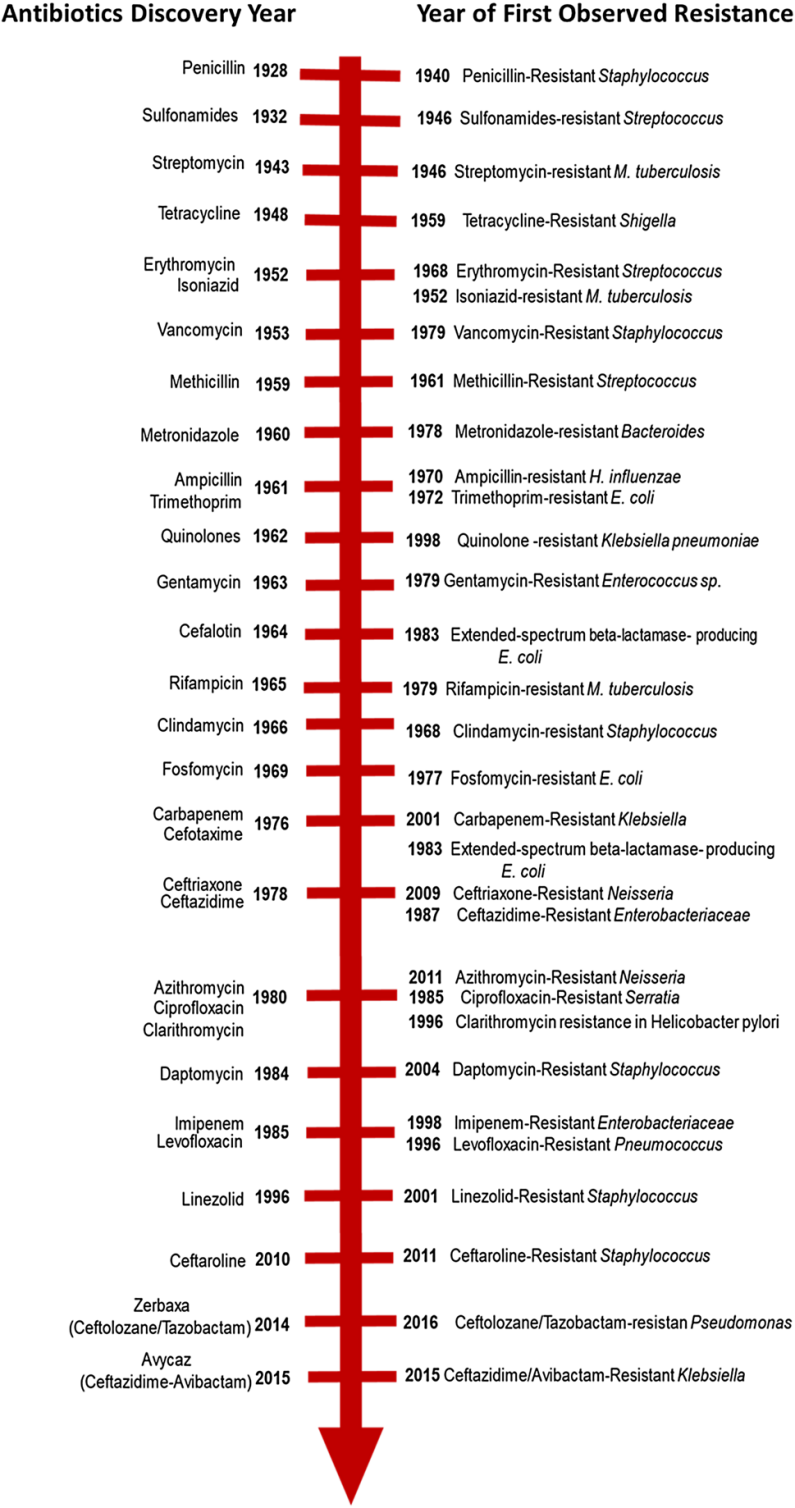
Timeline of antibiotics discovery and year of first observed resistance [15–33]

Antibiotics are among the most frequently prescribed drugs in modern medicine and have been used to treat bacterial infections since 1940s [7–9]. Bacterial resistance to antibiotics was first predicted by Alexander Fleming in 1945 during his Nobel Prize acceptance speech: “The time may come when penicillin can be bought by anyone in the shops. Then there is the danger that the ignorant man may easily under-dose himself and by exposing his microbes to nonlethal quantities of the drug make them resistant”. Many other factors may lead to resistance, including overuse of broad-spectrum antibiotics, and lack of early identification of causative pathogens and their antimicrobial susceptibility patterns. Additionally, heavy use of antibiotics in agriculture and intensive animal farming promote development of antibiotic resistance [10, 11]. These factors, together with poor infection control, are the leading culprits in the increasing spread of resistance [7, 12, 13]. While antibiotic resistance has mainly been a clinical problem in healthcare settings, recent studies show existence of resistant pathogens in both primary care patients and community settings [2]. This has been exacerbated by easy access to antibiotics in many developing countries, where one can go to any pharmacy and obtain any form of drug without prescription. Such practices either lead to overuse or underuse of broad-spectrum drugs, thereby increasing the risk of resistance and turn-over rates. Alarmingly, this has contributed to rapid development of resistance and rapid loss of effectiveness of new antibiotics, usually within five years of introduction into the market [14]. Figure 1 demonstrates the turn-over rates of various antibiotics from 1940 to 2015.

**Fig. 2:**
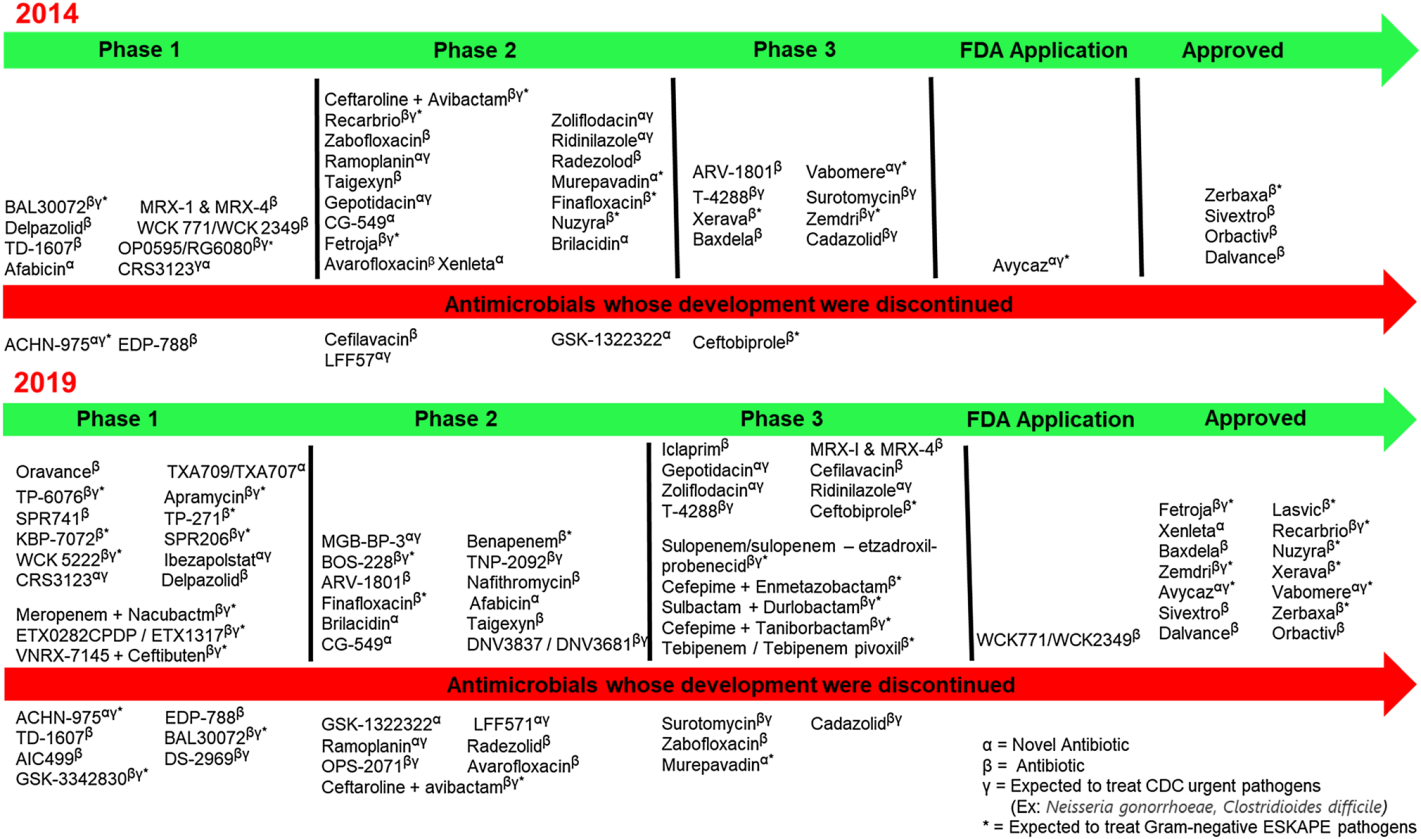
Antibiotic development pipeline from 2014 to 2019. As of December 2019, a total of 41 antibiotics were in development (15 in Phase 1 clinical trials, 12 in Phase 2, 13 in Phase 3, 1 submitted for FDA application), and 14 approved. It is estimated that only 60% of drugs that enter Phase 3 will be approved for treatment (pewtrusts.org). New antibiotic development involves time and resources and there are very few novel antibiotics under development. The declining number of antibiotics in the development pipeline, in part, reflects the challenges associated with its development. At the same time, bacteria that survives antibiotic treatment are spreading

Most bacterial pathogens utilize various resistance mechanisms to render antibiotics ineffective. These include the use of efflux pumps, inactivating enzymes, target modification, and microenvironment modifications [34]. These antibiotic resistance mechanisms pose serious challenges to the pharmaceutical industry in developing new drugs. The process of developing new antibiotics is time-consuming and extremely costly. As of December 2019, a total of 41 antibiotics were in development (15 in Phase 1 clinical trials, 12 in Phase 2, 13 in Phase 3, 1 submitted for FDA application), and 14 approved. It is estimated that only 60% of drugs that enter Phase 3 clinical trials will be approved. Figure 2 shows the list of antibiotics in the pipeline between 2014 and 2019 as well as those that have been discontinued. Given the mismatch between the rate at which bacteria develop resistance and the slow pace of new drug development, the world may soon run out of effective antibiotics. As a result, there is renewed interest in identifying potent new bioactive compounds with the hope to develop novel antibiotics that are less amenable to bacterial resistance.

### Developing potent novel drugs from plants sources

The use of medicinal plants in controlling diseases has been documented throughout the history of man. Traditionally, different parts of plants (leaf, stem, bark, root, fruit) have been used to treat, prevent, and control several diseases [35]. The World Health Organization (WHO) has prioritized the search for new antibacterial agents against multidrug-resistant ESKAPE pathogens (*Enterococcus faecium, Staphylococcus aureus, Klebsiella pneumoniae, Acinetobacter baumannii, Pseudomonas aeruginosa,* and *Enterobacter species*) [36]. These rapidly evolving pathogens are responsible for most of the cases of hospital-acquired infections globally [36]. Over the years, several medicinal plant extracts and secondary metabolites have been explored for their efficacy against these pathogens [37]. Some of these are:

- Different parts of *Adiantum capillus-veneris* and leaf extract of *Artemisia absinthium* have shown inhibitory effects against *E. faecium* and *S. aureus* [38].
- Leaf extracts of *Aloe ferox, Cynodon dactylon, Acacia nilotica,* bud *of Syzygium aromaticum,* and seed and leaf of *Theobroma cacao* were active against *Klebsiella pneumoniae* [39–41].
- Leaf extracts of *Mentha sp. a*nd *Aloe vera* and root of *Zingiber officinale* significantly inhibited *P. aeruginosa* growth [42].
- Root of *Piper longum,* stem of *Kalanchoe fedtschenkoi,* and fruit extract of *Martynia annua* were all found to be active against *A. baumannii*, [43–45].
- Leaf and seed extracts of *Dacryodes edulis* have activity *against E. cloacae* [46].
- Leaf extracts of *Ipomoea batatas* and *Hibiscus esculentus, leaf* and seed extracts of *Dacryodes edulis, bark of Azadirachta indica* have inhibitory effects against *E. aerogenes* [46].

In addition to their antibacterial properties, medicinal plants have also been used in traditional medicine for the treatment of both human and animal fungal diseases [47]. The increased use of antifungal agents in addition to the spread of multidrug-resistant fungi, and limited number of drugs available has precipitated an interest in new classes of antifungal drugs. Recent reports showed anti-fungal activities of several medicinal plants against different fungal species, including *Candida albicans*, Aspergillus species, Trichophyton species, Microscopium species, penicillium species, Fusarium species, Epidermophyton species, and *Rhodotorula ruba* [48]. Some of these plants are:

- Leaf extracts of *Eugenia uniflora, Psidium guajava, Curcuma longa, Piptadenia colubrina, Persea americana* showed activity against *C. albicans, C. dubliniensis, C. glabrata,* and *C. krusei* [49].
- Leaf extract of *Alibertia macrophylla* exhibits inhibitory effects against *Cladosporium sphaerospermum, C. cladosporioides, A. niger,* and *Colletotrichum gloeosporioides* [49].
- Leaf extract of *Piper regnellii* inhibits growth of *Trichophyton rubrum, Trichophyton mentagrophytes,* and *Microsporum canis* [50].
- Root extract of *Rubia tinctorum* was active *against A. niger, Alternaria alternaria, P. verrucosum,* and *Mucor mucedo* [51].
- Different parts of *Tithonia diversifolia* were active against *Microbotryum violaceum* and *Chlorella fusca* [52].
- Seed of *Cassia tora* showed inhibitory activity against *Botrytis cinerea, Erysiphe graminis, Phytophthora infestans, Puccinia recondita,* and *Pyricularia grisea* [53].
- Leaves and twigs of *Chamaecyparis pisifera* showed activity against *P. oryzae* [54].

The antiviral activities of medicinal plants have also been evaluated. The toxic side effects and ineffective response to the available antiviral drugs, especially in the wake of the coronavirus pandemic, has prioritized the development of potent agents to control deadly viral infections. Medicinal plants have been shown to possess potent antiviral agents with various activities against HIV, HBV, and several other viruses [55–59]. Exploring these plants and their bioactive metabolites will be a cost effective and secure way to develop new potent antiviral agents to combat viral diseases. Interestingly, 80% of the chronic Hepatitis B patients in China still rely on medicinal plants as primary treatment [60]. Some of these plants with antiviral activity showed similar or better efficacy against viruses than the available treatment options [60]. Among the reported medicinal plants with antiviral properties include:

- *Bulb of Allium sativum* L. has demonstrated potent antiviral activities against ADV-3, ADV-41, DENV, SARS-CoV-2, HSV-I and II, HCMV, H9N2, IBV, H1N1, CBV-3, ECHO, EV-71, HRV-2, HAV, MeV, PIV-3, VV, [61].
- Leaf of *Justicia adhatoda L.* was active against SARS-CoV-2, influenza virus, and HSV [60, 62].
- Rhizome of *Cyperus rotundus L.* inhibited SARS-CoV-2, HAV, HSV-I, and CVB [63, 64].
- Leaf of *Ocimum basilicum L.* was active against HIV-I, HSV, ADV-3, 8, 11, HVB, EV, and CVB-I [65–67].

Many of these plant extracts act by inhibiting viral replication, enhancing cellular immunity, inhibiting virus-cell attachment, inducing apoptosis of viral-infected cells, disrupting viral envelopes, inhibiting viral RNA and DNA synthesis, downregulating the expression of important host proteins, and inhibiting viral attachment to host cell surface [68].

Plants are rich in secondary metabolites and are a major source of chemical diversity, thus, may be promising sources of untapped potent antibacterial agents. Phytochemical analyses of some of these medicinal plants show different active groups, such as flavonoids, quinones, lignans, stilbenes, tannins, alkaloids, terpenes, polyphenolics, and coumarins [69], most of which are antibacterial in nature. For instance, phenol derivatives inhibit bacterial growth by either reducing the pH, increasing membrane permeability, or altering efflux pumping [70]. Phenolic compounds, one of the important secondary metabolites, have shown to act on many bacterial targets including cytoplasmic membrane damage, topoisomerase inhibition, NADH-reductase and ATP synthase inhibition [71]. Tannins have also been shown to induce bacterial membrane damage and metabolism inactivation [72]. Flavonoids, in turn may promote formation of extracellular complex soluble proteins and inhibit cell wall proteins as well as metabolism and DNA synthesis [73]. These mechanisms of action associated with plant secondary metabolic compounds make them promising agents to be harnessed to develop novel drugs to combat the growing problem of antimicrobial resistance.

**Fig. 3:**
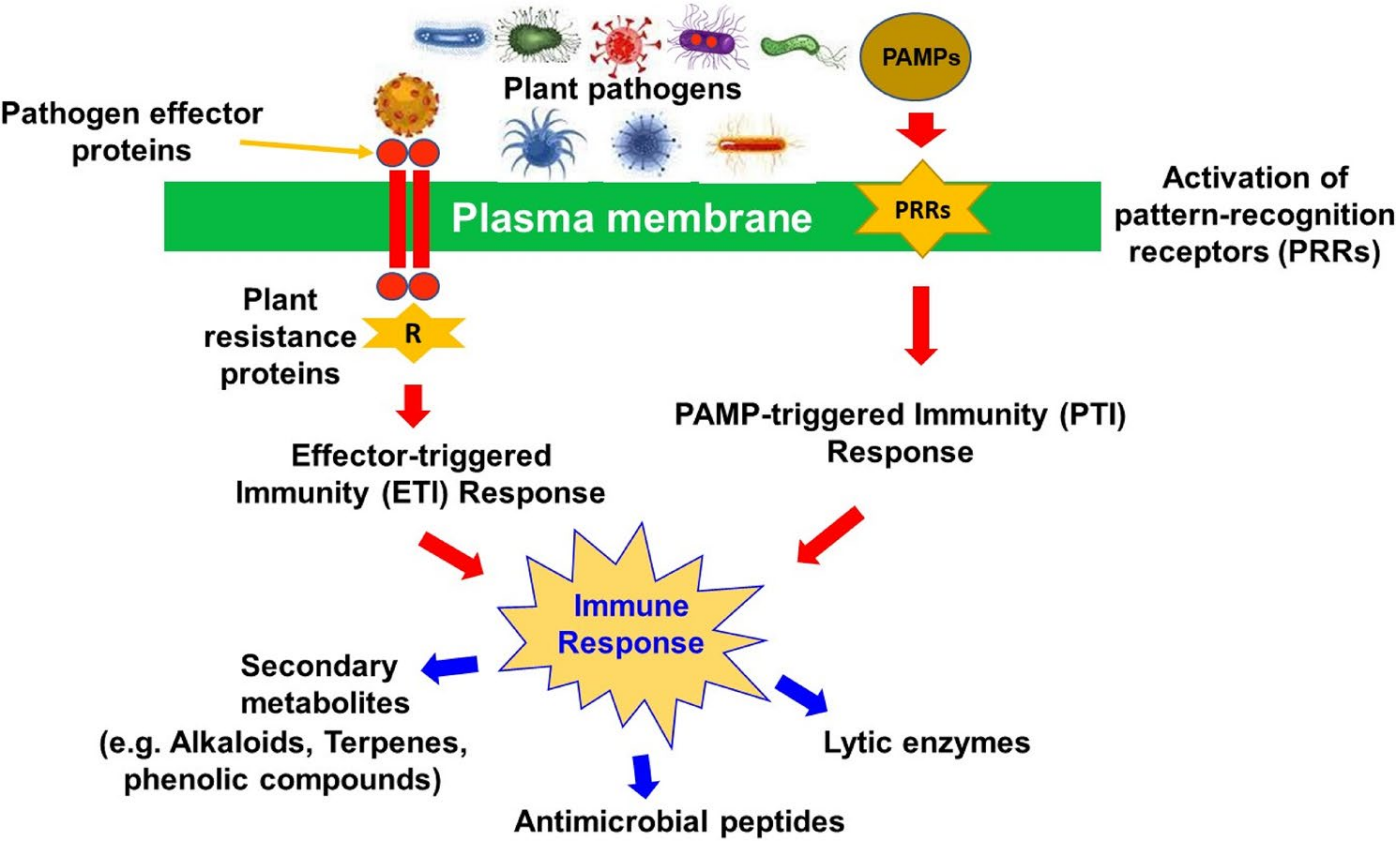
Plant immune response to pathogens. Bacteria are detected by either pathogen associated molecular patterns (PAMPs) or pathogen effectors: **I** The PAMPS activates the pattern-recognition receptors (PRRs) on the plant cell surface, which in turn activates a signaling cascade leading to PAMP-triggered immunity (PTI). **II** Pathogen effectors are recognized by plant resistance proteins, resulting in a hypersensitive response known as effector-triggered immunity (ETI). Together, these defense mechanisms result in the release of various secondary metabolites that ultimately kill the infecting pathogen. Given their novelty to human pathogens, these plant-derived antimicrobial secondary compounds can be harnessed to combat multidrug-resistant pathogens

Plant secondary metabolites are usually produced as defensive mechanisms against predators, plant pathogens, insects, and animals. During response to pathogens, surface receptors present on plants detect infecting agents by recognizing specific patterns and chemical motifs [74]. Plants detect bacteria using either pathogen associated molecular patterns (PAMPs) or pathogen effectors (Fig. 3). The PAMPS are sensed by pattern-recognition receptors present on plant cell surfaces, which in turn activates a signaling cascade leading to PAMP-triggered immunity, the primary immune response in plants [75]. Bacteria can, however, interfere with PAMP-triggered immunity by injecting effector molecules into the plant cell. These effectors are recognized by plant intracellular protein complexes such as the nucleotide-binding leucine-rich repeat receptors, resulting in a hypersensitive response known as effector-triggered immunity, the secondary immune response in plants [76, 77]. These mechanisms either limit pathogen entry, restrict pathogen propagation, or kill pathogens within the host plant cells. Once a pathogen is identified, plant cells also protect themselves by either reinforcing cell wall biosynthesis of lytic enzymes, producing secondary metabolites, or other pathogenesis related proteins [78].

Several bioactive compounds and their derivatives have been used as drugs for the treatment of different diseases, including cancer, hypertension, immuno-suppression, neurological diseases, fungal, viral and bacterial infections; some of which are either currently under clinical trials or already in the market [79]. Importantly, these compounds have demonstrated promising results in fighting the emergence of antibiotic resistant bacteria [72] and increasing the potency of old antibiotics through synergistic association, thus, preventing the development of resistance [80]. Some examples are:

- Berberine: an isoquinolone isolated from plants such as *Rhizoma coptidis.* Berberine is known to possess activity against methicillin-resistant *Staphylococcus aureus* (MRSA) by inhibiting adhesion to human gingival fibroblasts, an important step during biofilm development [81]. In addition to its ability to inhibit biofilm formation, several studies have reported a positive synergistic activity of berberine when combined with other antibiotics. For instance, addition of berberine to azithromycin and levofloxacin lowers its minimum inhibitory concentration by 50.0–96.9% [82], as well as decreases adhesion and intracellular invasion of MRSA [83].
- Piperine: a piperidine-type alkaloid was isolated from the *Piper species (Piper nigrum, Piper longum).* This compound has strong antimicrobial activity against both Gram positive and negative bacteria (*S. aureus, Bacillus subtilis, Salmonella sp and Escherichia coli) *[84] and acts as an efflux pump inhibitor in *S. aureus* when combined with ciprofloxacin [85]*.*
- Allicin: a sulfur-containing compound that is obtained from raw garlic (*Allium sativum*). Allicin has been shown to exhibit broad-spectrum antimicrobial activity against both Gram-positive and negative bacteria, including MRSA, *Streptococcus* spp., *E. coli*, and *Salmonella enterica* serovar Typhimurium [86]. Allicin acts through *S*-allylmercapto modification of thiol-containing proteins in bacteria, leading to reduction of glutathione levels, induction of protein aggregation, and inactivation of essential enzymes [86–88].
- Ajoene: another organosulfur found abundantly in oil-macerated garlic. Ajoene exhibits antibacterial activity against several Gram-positive and Gram-negative bacteria, including *H. pylori,* *Mycobacterium* species, however, its antimicrobial property was more observed in Gram-positives [89]. The mechanism of action of this compound is similar to that of allicin. The use of this compound to treat antibiotic resistant organisms is promising as it is now produced by total synthesis [90].
- Eugenol (4-allyl-2-methoxyphenol): a hydroxyphenyl propene, naturally occurring in essential oils from several plants belonging to the Lamiaceae, Lauraceae, Myrtaceae, and Myristicaceae families [91]. Several mechanisms of action of Eugenol has been reported, including inhibition of *Streptococci* biofilm and enterotoxin formation, disruption of *Salmonella typhi* cell membrane, and reduction of *S*. *aureus* toxin gene expression [92]. In addition, Eugenol has also been reported to inhibit production of bacterial virulence factors, such as violacein, elastase, pyocyanin [93].
- Resveratrol (3,5,4′-trihydroxystilbene) is a naturally occurring polyphenolic antioxidant that has received massive attention for its potential health benefits. It can be extracted from different plant species, such as grapevines, pines, bananas, beans, pomegranates, peanuts, and soybeans, The antimicrobial activity of resveratrol has not been fully studied. However, it exhibits antibacterial activity against several Gram-positive and Gram-negative foodborne bacteria by inhibiting gene expression [94]. Resveratrol also inhibits toxin production, biofilm formation, motility and interferes with quorum sensing in a wide range of bacterial, viral and fungal species [95].

These plant-derived metabolites could potentially be harnessed as novel drugs to combat antibiotic-resistant bacteria due to their natural origin with no history of prolonged exposure to human pathogens. To our knowledge, no resistance to plant-based compounds has been recorded to date. Moreover, secondary metabolites from plants have different active moieties and offers a repertoire of different activities that may be utilized against different bacterial targets [96]. Many cost-effective approaches are available to identify, quantify and characterize the bioactive plant compounds for further investigation as potential new drug molecules [97]. These include the use of spectroscopy, gas chromatography, high-pressure liquid chromatography, and thin-layer chromatography to provide improved extraction efficiency, yield, extraction time, selectivity, and sensitivity in quantitation [98, 99].

## Conclusion

Antimicrobial resistance is a major global health problem. This has been precipitated by rapid development and spread of resistant mechanisms resulting in loss of effectiveness of new antibiotics, usually within five years of introduction into the market [14]. As of today, no effective drug is available to reverse antibiotic resistance in bacteria. Several approaches have been undertaken to control bacterial resistance, including controlling antibiotic prescription, enhanced antimicrobial stewardship programs to improve antibiotic therapy, and developing new drugs. Another important approach, but less studied is to harness plant-based compounds. Plants are rich in several antimicrobial secondary metabolites and may be a rich source of potent drugs with a variety of chemical moieties that could target different resistant mechanisms in bacteria. Several plant species have already been reported to show potential antimicrobial effects against multidrug-resistant bacteria (Table 1). A deeper understanding of the mechanisms of action of these plant-derived compounds is needed. Harnessing secondary plants metabolites would be a cost-effective and innovative strategy to develop next generation novel antimicrobials and/or improve current antimicrobials to combat the emerging threat of antibiotic resistance, develop databases for plant metabolites, and their possible antimicrobial targets.

**Table 1:** Examples of plants with known activity against multidrug-resistant pathogens. These plant-based metabolites provide promising option to develop novel drugs against multidrug-resistant pathogens

Plant name and part	Extract type	Resistant bacteria	Evaluation method	Source, geographical location
*Moringa oleifera* **(Leaves)** *Metricaria recutita* **(Flowers)**	Water, Ethanol, Methanol	Clinical MDR, XDR, PDR isolates: *Escherichia coli* Klebsiella spp *P. aeruginosa* *Proteus mirabilis* *S. aureus* *S. epidermidis*	Microbroth dilution Disc diffusion	Farm in El-Fayoum governorate, Egypt [100]
*Scutellaria barbata * (***Herbs***)	Water extracts	Clinical MDR *Acinetobacter baumannii*	Disc diffusion, time-kill assays, murine lung infection model	Herb store in Kaohsiung City, Taiwan [101]
*Allexis cauliflora * (***leaves***) *Persea Americana * (***Stones***) *Entada Africana ****(bark)*** *Pentaclethra macrophylla *(***Bark***)	CH2Cl2/MeOH MeOH C4H8O2 Extracts	Kanamycin-resistant *E. coli AG100A*	Microbroth dilution	Different regions of Cameroon [102]
*Entada abyssinica * (***Leaves and roots***)		Clinical MDR *Klebsiella pneumoniae Kp55*		
*Pentaclethra macrophylla * (***Bark***)		Clinical MDR *Providencia stuartii-NAE16*		
*Alkanna tentoria* (***leaves***)	Aqueous, chloroform, ethanol and hexane extracts	*A. baumannii*, *E. coli*, *P. aeruginosa S. aureus*	Well diffusion	Charsadda region, Pakistan [103]
*Artemisia absinthium ****(Bark)***	Aqueous Ethanol Extracts	*Enterococcus faecium, Staphylococcus aureus*	Disc diffusion, time-kill assays	Sudhnoti district, Northern Pakistan [104]
*Martynia annua ****(bulk)***		*Enterococcus faecium,Staphylococcus aureus, Acinetobacter baumannii*	Disc diffusion, time-kill assays	
*Adiantum capillus-venaris ****(Bark)***		*Enterococcus faecium, Staphylococcus aureus*	Disc diffusion, time-kill assays	
*Zanthoxylum armatum**** (Bark)***		*Enterococcus faecium, Staphylococcus aureus*	Time-kill assays	
*Swertia chirata* **(Bark)**		*Staphylococcus aureus*	Disc diffusion, time-kill assays	
